# Allopurinol is the most common cause of DRESS syndrome in Hungarian patients

**DOI:** 10.1186/2045-7022-4-S3-P17

**Published:** 2014-07-18

**Authors:** Ágnes Kinyó, Katinka Ónodi-Nagy, Erika Varga, István Balázs Németh, Irma Korom, Rolland Gyulai, Lajos Kemény, Zsuzsanna Bata-Csörgõ

**Affiliations:** 1 University of Pécs, Department of Dermatology, Venereology and Oncodermatology, Hungary; 2 University of Szeged, Department of Dermatology and Allergology, Hungary; 3 University of Pécs, Department of Dermatology, Venereology and Oncoder, Hungary

## Background

Drug reaction with eosinophilia and systemic symptoms (DRESS) is a heterogenous group of severe adverse reactions to medications.

## Method

We have investigated the clinical and pathological features, and outcomes of DRESS presenting to our clinic from january 2002 to December 2012. Patients were selected as DRESS using the criteria of European Registry of Severe Cutaneous Adverse Reactions (RegiSCAR).

## Results

There were 77 total cases (24 male and 53 female; age range: 19-88 years; mean age 67 years). The most common culprit drug was allopurinol, followed by carbamazepine, lamotrigine, clindamycin and strontium ranelate. Indications for allopurinol therapy in all cases were asymptomatic hyperuricaemia. The time period from using the culprit agent to onset of the drug reaction ranged from 6 to 90 days. with a mean time of 28.4 days. The longest latency period (43.5 days) was for strontium ranelate. Four distinct patterns of cutaneous involvement were identified: a morbilliform, maculopapular exanthema (48 cases), an exfoliative erythroderma (21 cases), an urticated papular exanthem (4 cases) and an erythema multiforme-like reaction (4 cases). Pathologic changes observed were erythema multiforme, superficial spongiotic dermatitis with eosinophilia and lichenoid dermatitis. Impairment of liver and renal functions and blood dyscrasia were frequent complications. Most patients were treated with systemic corticosteroids. The mortality rate was 5.2%.

## Conclusion

High eosinophil count, atypical lymphocytes were poor prognostic factors in our patients with DRESS. Early diagnosis and prompt intervention therapy are essential.

